# Miscellaneous

**Published:** 1893-02

**Authors:** 


### MISCELLANEOUS.

### THE VENDETTA IN CORSICA.

The cross is a threat of death, and the Corsican who finds it drawn upon his door knows that he must look for no quarter, In decrees forbidding the carrying of arms in certain districts, exception is officially made in the case of persons notoriously *en état d'inimitie.* The vendetta neither sleeps nor knows where it may stop. It is not confined to two persons. The quarrels of individuals are taken up by whole families. Not even collateral branches are exempt, and women must take their chances with the men. Indeed, revenge is more artistically complete when the blow falls upon the beautiful and gifted. In 1856, one Joseph Antoine injured a girl named Sanfranchi. Thirty years passed and the story was forgotten, but on August 14, 1886, the nephew Sanfranchi encountered Antoine on perhaps the first occasion he had ventured far from his house. He shot the man down like a dog.

Threatened persons remain shut up for months or even years in their houses, built as all Corsican houses are, like a fortress. If they wish to go out for a moment to breathe the fresh air on the threshold, a scout goes before and reconnoiters. In the district of Sartene bands of armed men are sometimes met with in the road. It is a man *en inimitie* traveling from one village to another. The vendetta between the Rocchini and the Tafan resulted in the death of eleven persons and the execution of one of the principal criminals. In this extraordinary case two entire families took to the *marquis* and waged a guerrilla war upon each other; each in turn was assisted by gendarmerie, who had made disgraceful alliance with bandits in order to effect their arrests.

Contrary to custom, some of these bandits became brigands. As a rule persons outside their quarrel are never molested by them. They are merely outlaws. The Rocchini who was guillotined in 1888 (the first execution for many years) boasted that he was only twenty-two and had killed seven persons with his own hand. Confident of reprieve, he continued to regard himself as a hero until the day of his execution. When all hope was gone he sank into the most abject state of cowardice, which lasted until the end.

### APACHE ARROW POISON.

L. B. Hawks, recently in the U. S. Government Indian Service in Arizona, gives a description of the manner in which some of the braves in the Apache region prepare their deadly arrows. Although the Apaches have had little or no use for their poisoned weapons for years, still they, because of a tribal instinct, each summer go through a preparation of their arrow tips as carefully and methodically as though an old time war were at hand.

This work on the arrows is one piece of labor that the Indian brave will not leave to the squaws. He gathers a dozen or more rattlesnake heads and puts them in a spherical earthen vessel. With these he puts half a pint of a species of large red ant that is found in many parts of Arizona. The bite of this ant is more poisonous than that of a bee. Upon those he pours a bit of water, and then seals up with moist earth the lid of this vessel. He then digs a hole two feet deep into the ground, in which he builds a roaring fire and puts in some stones. When the interior of the hole and the stones are red hot he makes a place in the bottom for the earthen vessel and puts it in. About it and upon it he puts the coals and hot stones and upon the top he builds a fierce fire and keeps it up for twenty-four hours. Then he digs out his vessel, and, standing off with a long pole, he disengages the top and lets the fumes escape. The Indian insists that if the fumes should come in his face they would kill him. The mass left at the bottom of the vessel is a dark brown paste.

To test the efficacy of his concoction, Mr. Hawks has seen an Indian with his hunting knife make a cut in his leg, just below the knee, and let the blood run down to his ankle. Then taking a stick, he dipped it into the poison and touched the descending blood at the ankle. It immediately began to sizzle, as if it were cooking the blood, and the poison followed the blood right up the leg, sizzling its way, until the Indian scraped the blood off with the knife. The savage assured Mr. Hawks that had he allowed the poison to reach the mouth of the wound he would have been a dead man in twenty minutes.

---

An Ingenious Device.—Dogs are not partial to muzzles, but an artist recently invented a muzzle for his dog which in no way disconcerted the animal. He painted a representation of a muzzle over the dog's head so cleverly that all the policemen were deceived by it. The fraud was discovered by an old lady whose pug dog hated a muzzle so much that she allowed the animal to roam about without one. When the police captured her dog, the lady complained that the painter's dog went about without the customary head gear. The policeman assured her that the artist's dog was always muzzled, and was petrified with astonishment on learning that the muzzle was simply painted on the dog's head.

Will Our Sun Burn Up ?—Thousands of curious and ingenious theories have been brought forward to account for the fact that the sun although he has whirled his burning disc across the heavens for untold ages, continues to burn without his bulk, as far as we can tell, being lessened in the least. Some learned men believe that the sun is a monstrous ball of gas, but even a ball of gas of the size the sun is known to be would be entirely consumed in the course of a very few thousand years, to put it at the utmost limit. Others, again, tell us that the fires of Old Sol are kept up by the remains of wrecked worlds, which are constantly plunging into his great seas of gas. One of the most eminent astronomers of the age, in giving his opinion on this last conclusion, says that if a mountain range, or a section of a "wrecked world" 176 cubic miles in extent were to fall into the sun it would only be sufficient to maintain the present heat for a second.

Country Girls.—If your lives have fallen into some quiet, unpretentious place, do not complain that it is dull and commonplace, and that "there is nothing to live for here," as so many do. Why, there is no place on God's earth so bleak and barren, so quiet and lonely, so wind-swept and rain-beaten, but that there is a great deal to live for there, and when you have grown a little older you will see it with clear eyes ; and you will look back to the country village and wish—oh, how you will wish !—that you had been happy and content in that simple life. You will know, then, that it is nobler to live well a humdrum life than to wear out body and mind and soul in a fever of gaiety and frivolity, and to stretch out your empty hands always to something you cannot seize. Better to sing babies to sleep in the soft twilights that fold down over a cottage home than to loll in cushioned carriages and laugh at the brainless nonsense that may be whispered into your ears. And better, far better, to dwell for ever away from the lights, and the roar, and the temptations, and the sins of the city, with a clean heart and a pure soul than to let the city's passionate unrest creep into your pulses and set them to beating in a mad chase after—death.—*Farm and Home, Eng.*

OUTRAGES UPON NATURE.—No lady can be really well clothed if her dress outrages nature's intentions in the structure of the human frame. Such outrages are, a waist like a stove-pipe, shoes that compress the toes into a cramped mass of deformity, and, it might even be added, gloves that confine the hand till it looks little better than a fin; the foot is irredeemably ruined to the destruction of spring and grace in movement, and to no inconsiderable injury to health. No doubt the crumpled clump of deformity common from wearing modern abominations is a thing an ancient Greek would have shuddered at.—*Prof. Geo. F. Watts.*

Ladies talk much of visiting the dwellings of the poor in order to teach and preach to them the ordinary rules of sanitation ; let us hope the short skirt worn by the poorer classes may be a lesson of cleanliness and sanitation to the ladies themselves.

The Pittsburg *Dispatch*, in commenting on women's fashions in dress, thus hits off long skirts for street wear : Dark trimmings at the bottom of skirts are stylish now. No Pittsburg lady need be out of this fashion after walking two blocks !

"Now that I have my bran-new train,"
She said with joyous smile,
"I think I'll take a little walk
And clean the streets awhile."

